# Upright proton therapy for esthesioneuroblastoma: a single-institution experience

**DOI:** 10.3389/fonc.2024.1348291

**Published:** 2024-01-30

**Authors:** Konstantin Gordon, Igor Gulidov, Daniil Smyk, Alexey Semenov, Kirill Golubev, Alyona Lemaeva, Sergey Koryakin, Enar Jumaniyazova, Polina Vishnyakova, Irina Eremina, Timur Fatkhudinov, Andrey Kaprin

**Affiliations:** ^1^ Proton Therapy Department, A. Tsyb Medical Radiological Research Center - Branch of the National Medical Radiological Research Center, Obninsk, Russia; ^2^ Research Institute of Molecular and Cellular Medicine, Medical Institution, P. Lumumba People’s Friendship University of Russia, Moscow, Russia

**Keywords:** esthesioneuroblastoma, proton therapy, radiation therapy, reirradiation, brain invasion, upright position

## Abstract

**Aim:**

This study presents an analysis (efficacy and toxicity) of outcomes in patients with esthesioneuroblastoma after pencil beam proton therapy with a fixed beamline in the upright position.

**Background:**

Esthesioneuroblastoma (ENB) is an extremely rare tumor of sinonasal area located in critical proximity to vital structures. Proton therapy (PT) is often considered the optimal radiation treatment for head-and-neck tumors, although of limited availability. Upright PT delivered using fixed pencil beamline and rotating chair is a fairly promising option.

**Methods:**

This is a single-center experience describing the outcomes of PT in 14 patients with ENB treated between January 2016 and October 2022; half of the cases had a history of previous irradiation. The therapy was applied using a fixed pencil beamline with 6D-chair for positioning. The median dose was 63 GyRBE (total range 48–70 GyRBE; based on 1.1 RBE multiplier for protons) with 2.0 GyRBE per fraction. The mean gross tumor volume was 109.5 cm^3^ (17.1–257.7 cm^3^). Patient demography, pathology, treatment parameters and toxicity data were analyzed. Radiation-induced reactions were assessed according to the Common Terminology Criteria for Adverse Events (CTCAE) v 4.0.

**Results:**

The median follow-up time was 28 months. The 1- and 2-year locoregional control rates constituted 100% and 88.9%, respectively; the median duration of local control was 52 months. The 1- and 2-year progression-free survival (PFS) rates constituted 92.9% and 75.0%, respectively; the median PFS duration was 52 months. The 1- and 2-year overall survival (OS) rates constituted 92.9% and 84.4%, respectively. Two patients died of non-cancer-related causes (coronavirus-induced pneumonia) and 1 patient died of tumor progression. All patients tolerated PT well without any treatment gaps. Serious late toxicity reactions included glaucoma in 1 patient and cataract in 2 patients, in over half a year since irradiation.

**Conclusion:**

PT with upright design of the unit affords promising outcomes in terms of disease control and toxicity rates in ENB, a sinonasal tumor of complicated localization.

## 1 Introduction

Esthesioneuroblastoma (ENB), also known as olfactory neuroblastoma, is a tumor of neuroectodermal origin that develops from olfactory receptor cells in the nasal cavity (1), wherefrom it tends to invade the adjacent areas of pterygopalatine fossa, skull base and sinuses. ENBs are rare neoplasms accounting for only 3–6% of all tumors of the nasal cavity, which limits the patient recruitment for randomized trials and interferes with the development of a uniform treatment strategy (1, 2). The treatment is further complicated by unspecific symptoms and accordingly a delay in medical attendance and proper diagnosis; hence the prevalence of advanced cases with locoregional and distant metastases (3).

By the spread of the primary disease, ENBs are classified into 4 stages according to the Kadish staging system: A, the tumor is confined to nasal cavity; B, the tumor spreads to paranasal sinuses; C, the tumor invades skull base, pterygopalatine fossa; D, distant metastases are present. Clinical decisions in ENB are typically based on Kadish stage (4), importantly also accounting for the Hyams morphological grading scheme (5). The treatment mainstay for ENB is a multimodal approach using surgery, irradiation and chemotherapy (6).

Radiation therapy (RT) along with surgery is used as curative treatment for ENB in stage A patients and some stage B patients. The 5-year survival rates with this approach reach 29–63% (4, 7, 8). For more common stages C and D, RT is used as neoadjuvant and adjuvant options. Combining RT with chemotherapy potentially can improve survival rates and reduce the probability of relapse (8).

RT for ENB is liable to multiple treatment-related risks; the factors include tumor localization in the nasal cavity, invasion into skull base, the need for higher doses (66–70 Gy) and the proximity to critically vulnerable neural structures (brain stem, optic nerves, chiasm, etc.). Only high-precision RT techniques should be used to alleviate the risks of severe late toxicity (9, 10). In this regard, proton therapy (PT) is particularly promising due to its favorable dose distribution properties that alleviate the radiation burden on risk organs located close to the tumor.

Since 2016 PT has been established as a standard radiation treatment for patients with ENB at Tsyb Medical Radiological Research Center in Obninsk. Here we present experience of ENB treatment with horizontal pencil proton beam in the upright patient position (Prometheus, JSC Protom).

## 2 Materials and methods

The study included 14 patients (pts) with morphologically confirmed diagnosis of ENB receiving a course of PT within the period from January 2016 to October 2022. All patients were approved for inclusion in a retrospective analysis by the Ethical Review Board at the Tsyb Medical Radiological Research Center. Informed consent was waived due to the retrospective nature of the study and anonymous use of the evidence. The patient data are given in **Table 1**.

**Table 1:** Patients’ and tumors’ characteristics.

Indicator	Number
**Median age**	53 (48- 59)
Sex	
Male	4 (28.6 %)
Female	10 (71.4 %)
Hyams grade	
Grade II	8 (57.1 %)
Grade III	6 (42.9 %)
Kadish stage	
A	1 (7.1 %)
B	5 (35.8 %)
C	8 (57.1 %)
Stage (TNM)	
I	1 (7.1 %)
II	1 (7.1 %)
III	2 (14.3 %)
IVа	4 (28.6 %)
IVb	6 (42.9 %)
Tumor origin	
Nasal cavity	9 (64.3 %)
Paranasal cavity	2 (14.3 %)
Other	3 (21.4 %)
Brain invasion	
Yes	5 (35.7 %)
No	9 (64.3 %)
Treatment history	
No	2 (14.3 %)
RT	1 (7.1 %)
Surgery	4 (28.7 %)
Surgery + RT	2 (14.3 %)
Surgery + CTX	1 (7.1 %)
Surgery + RT+ CTX	3 (21.4 %)
RT + CТX	1 (7.1%)

*CTX, chemotherapy; RT, radiation therapy.

PT was performed using a fixed horizontal beam of intensity-modulated protons with the patient sitting in a 6D-movable 360° rotating chair (upright position) (11). A standard immobilization device (thermoplastic mask) was used to fix the patient’s position. The patient’s positioning was guided each session using built-in cone-beam computed tomography (CB-CT) (12).

The target volumes were delineated as recommended by the international consensus guidelines for head-and-neck tumors (13, 14). For lymph collectors of the neck, the irradiation volumes were also selected in accordance with specific recommendations (15, 16). The contours of target volumes and risk organs were determined based on CT images obtained during simulation, co-registered with magnetic-resonance images (MRI). The target volume included gross tumor volume (GTV) with a margin of 5 to 10 mm (considering anatomical barriers and organs at risk) to obtain the clinical tumor volume (CTV). The margin to the planned tumor volume (PTV) was 3 mm. All patients underwent CT control (typically on 10^th^ and 20^th^ fractions) in order to identify and correct errors coming from inflammation and tumor response, as charged particles are sensitive to density changes. The treatment data are given in **Table 2**.

**Table 2:** Proton therapy characteristics.

Treatment data	Number
**Mean total dose (GyRBE)**	63 (48-70)
RT intention	
RT only	9 (64.3 %)
Postoperative RT	5 ( 35.7 %)
RT course	
Primary RT	7 (50%)
Repeated RT	7 (50%)
**Median time from previous RT (years)**	7.3 (3-17)
**Median previous dose (Gy)**	62 (50-70)
**Median GTV volume (cm^3^)**	109.5 (17.1–257.7)

*GTV, gross tumor volume; RBE, relative biological efficacy; RT, radiation therapy.

The relative biological effectiveness (RBE) of the protons was accepted as 1.1. Restrictions on risk organs were set considering the RBE according to the QUANTEC group recommendations. The doses were delivered using a conventional regimen, with 2 GyRBE per fraction.

The total doses were selected in accordance with medical histories: 60–70 Gy for primary cases and 48–60 Gy for reirradiation. The PT dose was prescribed to the PTV with the aim of at least 95% coverage, but in the case of meeting OAR limits, dose constraints prioritized PTV coverage. Doses for reRT cases were chosen based on the reserve of the series OARs, and time from prior RT. We used single-field optimized PTV-based plans, usually generated with 4–5 fields.

**Figure 1:**
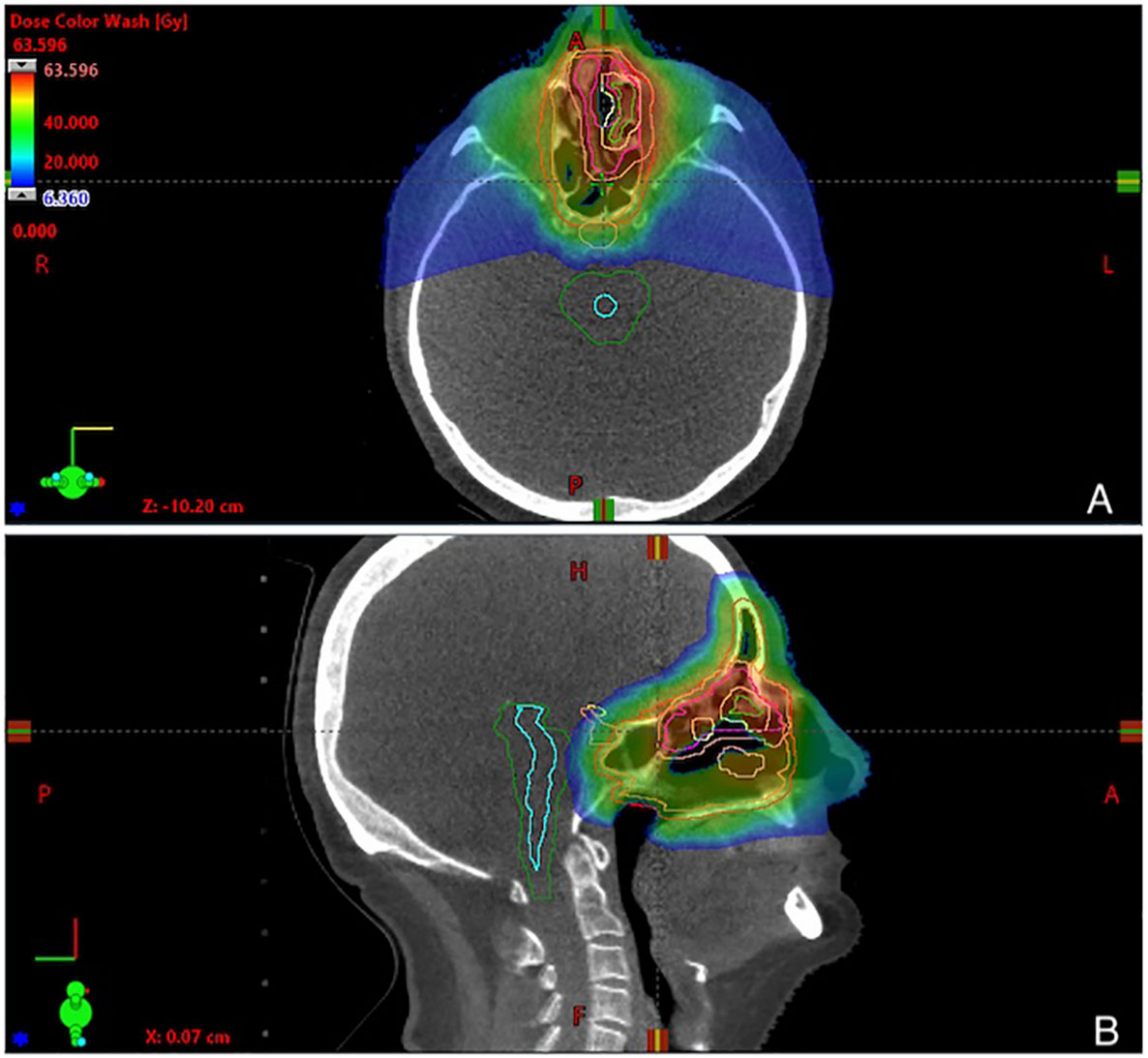
Representative 3-fields plan of proton therapy, showing dose distribution in **(A)** axial and **(B)** sagittal view. Physical doses are given in a dose legend **(A)**.

Representative plan of PT is shown in **Figure 1**. Treatment planning system ProtomTherapyPlanner ver. 2.14 (JSC Protom) was used.

All patients were examined before the start of the treatment, then every 3 months during the first year after the end of treatment and subsequently at 6 months intervals. The control examination after the course of PT included contrast-enhanced MRI (or CT in cases of MRI contraindication), ultrasound scan of cervical lymph nodes and positron-emission CT with glucose if required. The toxicity was assessed using CTCAE v 4.0 criteria.

The follow-up time was calculated from the end of the treatment to the last clinical assessment. Local control was defined as the absence of tumor growth in the irradiated area. Progression-free survival was defined as the absence of locoregional or distant progression. The overall survival (OS) was calculated from the end of PT to the last visit or date of death. Statistical analysis was carried out using StatTech v3.1.8 (Stattekh LLC). The survival curve was built using the Kaplan-Meyer method. An adjusted *p*-value <0.05 indicated a statistically significant difference; *p*-values ≥0.05, but <0.1 were noted as tendencies. No stratification by disease or treatment parameters was made due to the small size of the cohort.

## 3 Results

**Figure 2:**
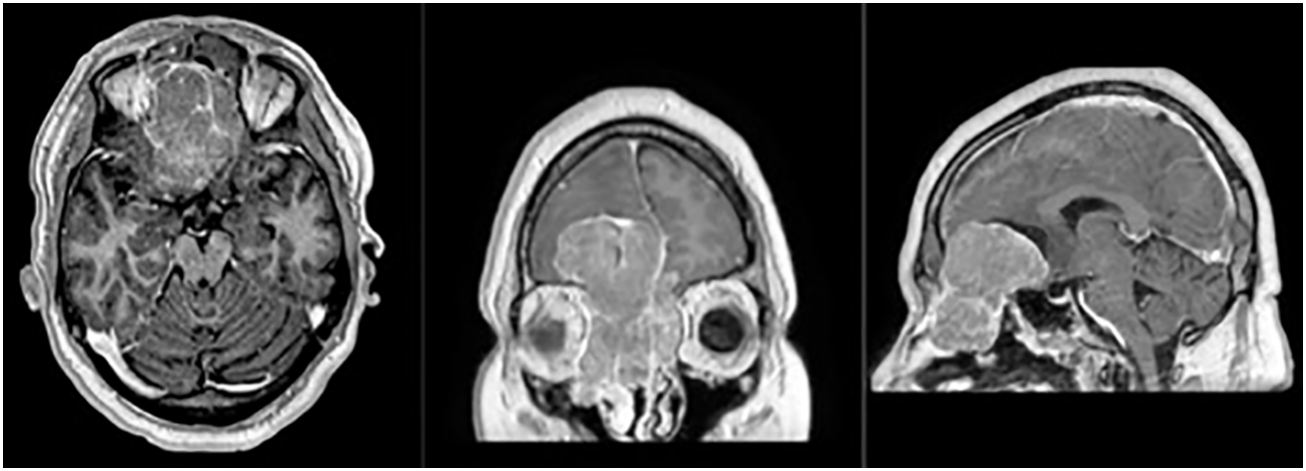
Magnetic-resonance (T1 sequence with contrast enhancement, axial, frontal and sagittal views) of the patient with Kadish C tumor stage, with a massive frontal lobes invasion.

The patient and PT data for 14 pts with ENB included in the study are listed in **Tables 1**, **2**. The mean age of participants was 53 years (range 48–59 years). Most of the tumors were located in the nasal cavity (64.3%) and paranasal sinuses (14.3%). Most of the patients had locally advanced disease at the time of the treatment, with intracranial invasion observed in 35.7% of the cases (**Figure 2**).

Two patients had no previous treatment history, 4 pts had surgery, 1 pt received RT only, 2 pts received surgery and RT, 1 pt received surgery with adjuvant and neoadjuvant chemotherapy, 1 pt received PT followed by chemotherapy and 2 courses of chemoembolization, 3 pts had surgery, and also received radiation and chemotherapy. Thus, 7 out of 14 pts underwent reirradiation.

**Figure 3:**
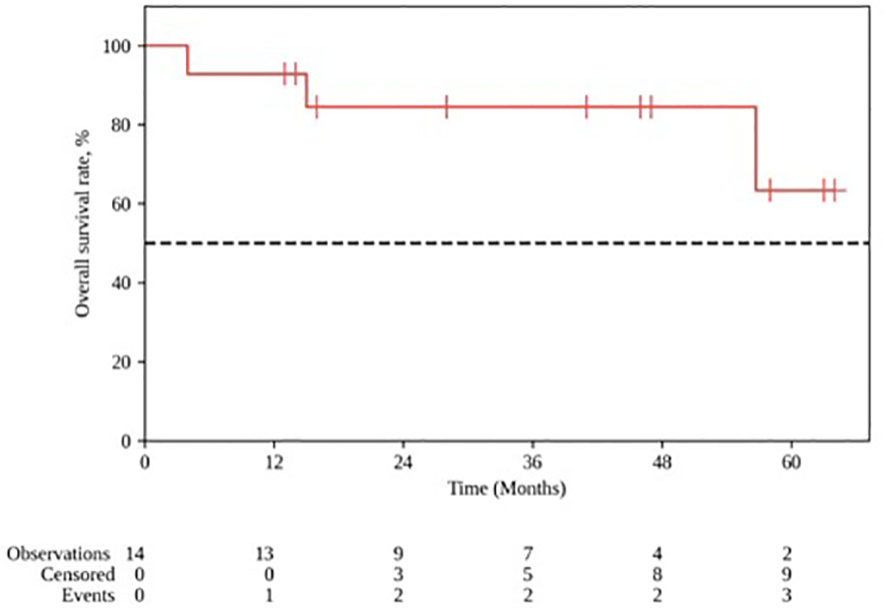
Overall survival after PT for ENB. Kaplan-Meyer plot.

**Figure 4:**
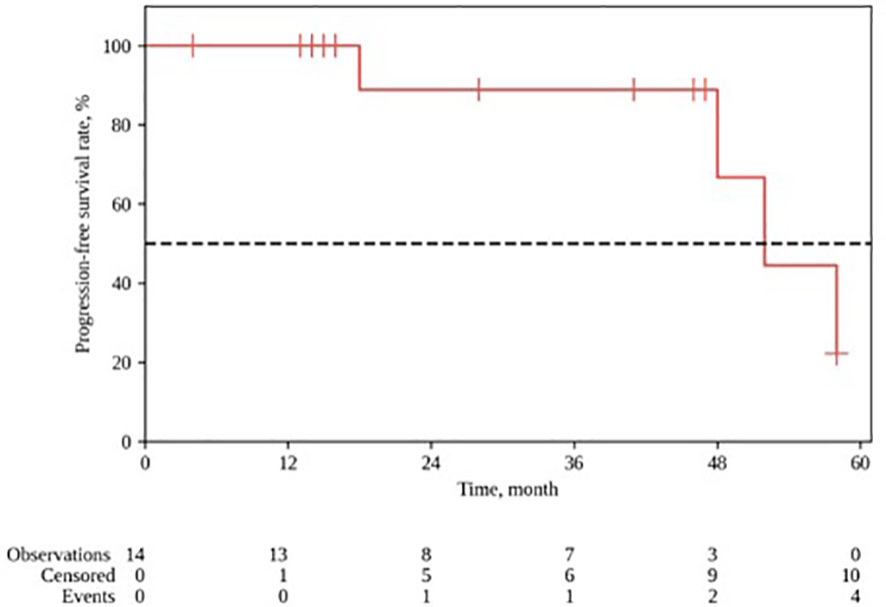
Progression-free survival after PT for ENB. Kaplan-Meyer plot.

The median follow-up for the group was 28 months (range 4–64). The 1- and 2-year locoregional control rates were, respectively 100% and 88.9% (95% CI: 43.3–98.4); the median local control duration was 52 months (95% CI: 43.9–60.0). The 1- and 2-year progression-free survival (PFS) constituted, respectively, 92.9% (95% CI: 59.1–99.0) and 75.0% (95% CI: 40.3–91.3); the median PFS was 52 months (95% CI: 18–∞). The 1- and 2-year OS rates constituted, respectively, 92.9% (95% CI: 59.1 – 99.0) and 84.4% (95% CI: 50.4–95.9) (**Figures 3**, **4**).

**Figure 5:**
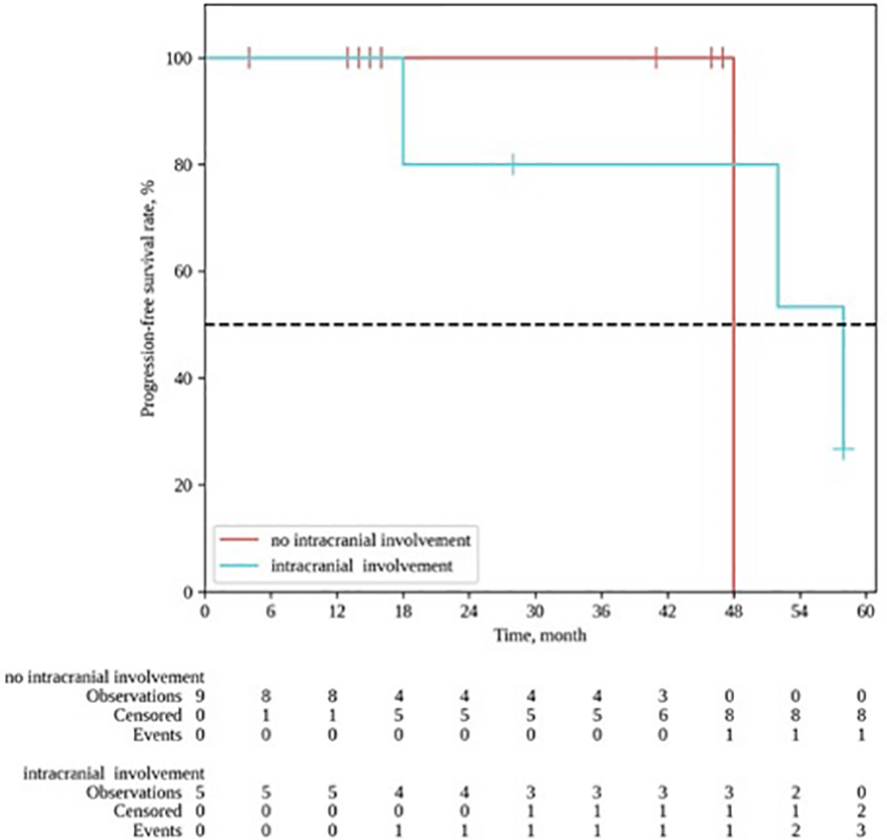
Locoregional control in patients with (blue line) / without (red line) frontal lobe invasion. Kaplan-Meyer plot.

In the follow-up, disease progression was recorded in 4 pts, including 3 pts with local (in-field) progression and 1 pt with lymph node metastases. Due to inoperability of the local relapses, 2 pts received systemic chemotherapy and 1 pt received chemoembolization, and 1 pt with regional progression underwent extended unilateral cervical lymphadenectomy. It should be noted that in all 4 cases of progression, the patients had stage IVa-b tumor process at the time of PT, and 3/4 pts had intracranial invasion appearing to interfere with the locoregional control after RT (p= 0.09) (**Figure 5**). No other significant factors influencing the treatment outcome were revealed. Two patients died due to non-cancer reasons (COVID-19 infection), and 1 pt died of disease progression with severe brain edema.

Early toxicity reactions were assessed for the entire cohort (14 pts, 100%). In 6 pts (42.8%) the early toxicity was limited to grade 1, in 7 pts (50.0%) it was grade 2, and 1 pt had acute toxicity of grade 3. The reactions, mostly local, included dermatitis (85.7%), mucositis (57.1%), conjunctivitis (50.0%) and xerostomia (28.5%).

Late toxicity events were recorded in 11 pts (78.5%). Specifically, 9 pts (64.2%) had grade 1-2 reactions including nasal cavity mucosa atrophy (64.2%) and in-field post-radiation fibrosis (35.7%). Also, 1 pt developed glaucoma and 2 pts developed radiation-induced cataract within 6–12 months after the treatment.

## 4 Discussion

Sinonasal esthesioneuroblastomas (ENBs) are rare malignant neoplasms of the head-and-neck region. The lack of uniform clinical approaches for ENBs is due to their rarity. In addition, the principle of clinical decision-making on the basis of tumor morphology plus localization might not be well applicable to ENBs, as these tumors may combine certain properties of neuroendocrine tumors (due to their origin from olfactory neuroepithelium) with those of squamous cell carcinomas. Additional difficulties arise from the established clinical classification for ENBs, which is different from the TNM staging system, and malignancy grade determination for these tumors, which also differs from the typically used grading system.

The default first-line option for ENB is surgery, but, given the tumor localization, curative surgery for ENB is often dismissed as crippling or technically unfeasible. The use of adjuvant modalities, notably a range of chemotherapies from platinum monotherapy to cyclophosphamide, decarbazine, etoposide, vincristine, etc., in various combinations over decades, produced extensive clinical data indicating the doubt of significant benefits of chemotherapy in ENB (8, 17).

The main clinical strategies envisioned for ENB account for its dual nature of neuroendocrine tumor and squamous cell head-and-neck carcinoma by using both the Kaddish staging system and Hyams histological grading. The local Kadish stage A-B and Hyams grade I-II are subject to monotherapy ― either surgery or RT. By contrast, locally advanced tumors of Hyams grade III-IV require a combination of surgery, chemotherapy and RT (optionally a combined chemoradiotherapy) (1). In such cases, RT can be considered as an alternative to surgical treatment (18).

The choice of irradiation method for ENB is extremely important, since the tumor is localized in the facial part of the skull packed with vital structures and also cosmetically important. With clear indications for RT, as ENBs are radiosensitive tumors (19), the risks of radiation complications should be addressed scrupulously.

PT is a generally accepted irradiation method that allows significant reduction of the dose to surrounding tissues, especially relevant for head-and-neck tumors (20). However, its use is limited by complexity, big size and high costs of the equipment. The upright position design of the unit levels some of these disadvantages, making the treatment more accessible for clinical practice while maintaining its quality (21).

Our study enrolled 14 patients who underwent PT for local and locally advanced ENB in upright position with an active scanning beam. The limitations of our finding include small size of the cohort, as well as its considerable heterogeneity. However, most of the published clinical evidence for ENB has similar limitations of small studies. Of note, even the largest analysis of ENB treatment for more than 900 patients provides no detailed data on RT outcomes (17).

The median age of our patients was 53 ( ± 10 years), which is typical for ENB (8). The median total radiation dose was high, amounting to 63 GyRBE. The median survival constituted 52 months. The 1- and 2-year locoregional control rates constituted 100% and 88.9%, respectively. At the time of this analyses, 11/14 pts remain under observation. Three patients died; in 2 cases the death was related to the consequences of coronavirus infection and 1 pt died from intracranial progression.

As demonstrated by Wang et al., intracranial extension is not an adverse prognostic factor in ENB treatment (22). In our cohort, about one-third of the patients had frontal lobe invasion at the time of the treatment. However, we observed a tendency towards worse treatment outcomes in cases of frontal lobe invasion (*p* = 0.09); in addition, the local control rates constituted 40.0% vs 88.9% for patients with and without intracranial invasion, respectively.

At the same time, such key parameters of the disease as Hyams malignancy grade, Kadish prevalence stage and the presence of regional metastases showed no significant effect on the local control in ENB (23), although this result may reflect the relatively short follow-ups and the issue should be additionally addressed for longer observation periods.

As demonstrated in some studies, PT not only has dosimetric advantages, but also favorable profiles of treatment-related toxicity (24). In our study, acute radiation toxicity was typically represented by grade 1-2 local mucositis and grade 1-2 dermatitis. The sinus area is one of the most difficult locations to plan and administer irradiation. PT with an active scanning beam is particularly challenging due to multiple density transitions (air-bone-soft tissue) in a relatively small volume, as well as changes in the density and size of the mucous membrane due to extensive inflammatory reaction. We used the strategy of 2 verification cone-beam CT scans after each 20 GyRBE, which allowed us to decrease the potential set-up and density errors and avoid late toxicity, especially from visual structures.

Late complications encountered for the cohort included grade 3 dry mucous membrane in 1 pt, hypoosmia in 2 pts and cataract in 2 pts. Also, 2 pts noted prolonged swelling of the nasal mucosa; 1 pt was diagnosed with ocular melanoma shortly after treatment, which required enucleation.

One of the earliest reports on PT in sinonasal ENB was published by Nishimura et al. in 2007. The authors presented clinical outcomes for 14 patients after PT on a 235 MeV cyclotron with a gantry system, with a dose of 2.5 GyRBE/daily and a total dose of 65 GyRBE. Five-year OS and PFS constituted, respectively, 93% and 84%. The authors encountered acute toxicity grade 1-2 and late toxicity grade 3, and no higher grade radiation toxicity reactions throughout the observation period (25).

Later on, a clinical experience of PT for sinonasal ENBs in a small cohort of 13 patients was reported by researchers at the University of Florida. The treatment was carried out with a passive scattered proton beam, in hyperfractionation mode, 1.2 GyRBE per fraction, which enabled safe delivery of 64.8–74.4 GyRBE doses. In this study, 10 of 13 recipients survived for at least 35 months; the acute toxicity was low and regressed within 4 weeks after the end of irradiation (26).

In 2015, a small experience of PT in 8 patients aged 4 to 21 was presented by Massachusetts General Hospital. Four-year OS in the group was 87.5%. It is important to note that 4 pts (half of the cohort) developed endocrine dysfunctions; other complications included retinopathy in 2 pts and grade III ophthalmopathy in 1 pt (27). The higher tissue radiosensitivity in children compared to adults requires special consideration when planning PT (28).

A cohort of 42 patients described by Nakamura et al. in 2017 can be considered the largest experience of PT for ENB. The authors noted a weak relationship of treatment outcomes to Kadish stage. Thus, 5-year OS rates were 100% and 76% for Kadish stages A and C, respectively, although the difference was statistically non-significant. Treatment failures were primarily due to distant or regional progression (48%) and only 10 pts developed local recurrence. Moreover, age under 50 was a significant favorable factor, even in cases of tumor progression. In contrast to the experience of PT at other centers including ours, the study encountered acute grade 3 reactions including mucositis in 4 pts and dermatitis in 1 pt. Visual complications grade 3-4 emerged in 4 pts; also, 1 pt with Kadish stage C developed grade 4 liquorrhea. Another important note was the lack of effectiveness of adding chemotherapy in Kaddish stage C (10).

In 2018, the University of Heidelberg presented experience with IMRT and carbon-ion therapy (CIT) in a heterogeneous group of 17 patients with primary and recurrent tumors, including 4 cases of re-irradiation, which was identified as a factor for a worse prognosis. In 13 patients without a history of RT, 4-year OS was 100%. The most common radiation toxicity reaction in this cohort was asymptomatic cerebral edema (30%) (29). Similarly with our study, 2 pts died of intracranial tumor growth. A history of RT had no effect on the outcomes probably due to the long interim between the courses (median gap 7.3 years).

Another study of 2018 enrolled 21 patients receiving CIT for T_4_ ENB in Japan. Three-year OS and LC rates were, respectively, 88.4% and 83%; 3 pts developed severe ophthalmopathy grade 4 (30).

One of the most recent studies on PT in ENB, published by Chang et al. in 2022. assessed post-operative IMPT in 15 patients with ENB; the doses constituted 60–72 GyRBE (median 68 GyRBE). At a median follow-up of 20 months, 2-year OS and relapse-free survival rates were, respectively, 88% and 83%. Severe complications encountered in this study included single cases of brain tissue necrosis and sinus obstruction (31).

In general, due to the rarity of ENBs, no optimal radiation treatment parameters for these tumors have been determined in clinical trials. Importantly, 50% of patients the studied cohort received reirradiation with protons. Thus, PT afforded positive clinical outcomes even for already treated and relapsed cases of ENB. A recent large multi-institutional analysis features PT as an effective and safe option of radiation treatment for tumors of sinonasal localization (including ENB) regardless of tumor morphology (32). Moreover, for sinonasal tumors, repeated irradiation with protons and/or carbon ions allows radiation toxicity risk minimization as compared with other protocols (33).

Upright PT is an old technology almost suspended from clinical use due to the lack of reliable means for precision patient positioning at the beginning of the proton era. With new technological advances in the image guidance, patient positioning, beam delivery, etc., the approach is gaining a renewed interest (21). Our own clinical practice comprises a positive experience of upright PT for similarly complicated tumors including skull base chordomas and chondrosarcomas (34).

Apart from small size and heterogeneous treatment history of the cohort. The second is its retrospective design of the study. All abovementioned studies are heterogeneous in treatment criteria, outcome assessment. This variability affects the direct comparability of the results. Still, we consider it important to report the outcomes for the state-of-the-art upright PT in ENBs due to their pathogenetic distinctiveness combined to rarity and limited knowledge about optimal management for these tumors.

## 5 Conclusion

The study provides a unique example of upright PT for sinonasal ENB. The outcomes indicate acceptable effectiveness and safety of the treatment independently of irradiation history. Accordingly, the treatment can be considered as a strong alternative to gantry PT in ENB.

## Data availability statement

The raw data supporting the conclusions of this article will be made available by the authors, without undue reservation.

## Ethics statement

This study was approved by the local ethical committee and the institutional review board of A. Tsyb Medical radiological research center—branch of the National medical research radiological center of the Ministry of Health of Russia, including waver of informed consent due to its retrospective nature. All procedures were performed following the ethical standards of the responsible committee on human experimentation and with the Helsinki Declaration of 1964, as revised in 2013.

## Author contributions

KoG: Conceptualization, Validation, Writing – original draft, Writing – review & editing. IG: Conceptualization, Supervision, Writing – review & editing. DS: Data curation, Writing – original draft. AS: Data curation, Formal analysis, Software, Validation, Writing – original draft. KiG: Data curation, Investigation, Methodology, Writing – original draft. AL: Data curation, Formal analysis, Resources, Writing – original draft. SK: Resources, Supervision, Validation, Writing – review & editing. EJ: Data curation, Formal analysis, Validation, Writing – original draft. PV: Formal analysis, Investigation, Supervision, Writing – review & editing. IE: Project administration, Resources, Supervision, Writing – review & editing. TF: Funding acquisition, Project administration, Resources, Writing – review & editing. AK: Funding acquisition, Project administration, Resources, Writing – review & editing.
